# Synaptogenesis Is Modulated by Heparan Sulfate in *Caenorhabditis elegans*

**DOI:** 10.1534/genetics.118.300837

**Published:** 2018-03-20

**Authors:** María I. Lázaro-Peña, Carlos A. Díaz-Balzac, Hannes E. Bülow, Scott W. Emmons

**Affiliations:** * Department of Genetics, Albert Einstein College of Medicine, Bronx, New York 10461; † Dominick P. Purpura Department of Neuroscience, Albert Einstein College of Medicine, Bronx, New York 10461

**Keywords:** C. elegans, heparan sulfate, neurexin, neuroligin, Proteoglycans, synapse formation

## Abstract

The nervous system regulates complex behaviors through a network of neurons interconnected by synapses. How specific synaptic connections are genetically determined is still unclear. Male mating is the most complex behavior in *Caenorhabditis elegans*. It is composed of sequential steps that are governed by > 3000 chemical connections. Here, we show that heparan sulfates (HS) play a role in the formation and function of the male neural network. HS, sulfated in position 3 by the HS modification enzyme HST-3.1*/*HS 3-*O*-sulfotransferase and attached to the HS proteoglycan glypicans LON-2/glypican and GPN-1/glypican, functions cell-autonomously and nonautonomously for response to hermaphrodite contact during mating. Loss of 3-*O* sulfation resulted in the presynaptic accumulation of RAB-3, a molecule that localizes to synaptic vesicles, and disrupted the formation of synapses in a component of the mating circuits. We also show that the neural cell adhesion protein NRX-1/neurexin promotes and the neural cell adhesion protein NLG-1/neuroligin inhibits the formation of the same set of synapses in a parallel pathway. Thus, neural cell adhesion proteins and extracellular matrix components act together in the formation of synaptic connections.

## Abstract (precis)

The nervous system relies on synapses to transmit information between neurons and thereby direct behavior, but how the correct synaptic connections are genetically specified is poorly understood. By genetically ablating enzymes that modify heparan sulfate...

BEHAVIORS are the result of a combination of signaling pathways coordinated at various cellular and tissue levels. Male mating is the most complex behavior of *Caenorhabditis elegans*. This behavior is governed by 144 neurons and 64 muscles in the posterior part of the male worm that are extensively interconnected to each other resulting in ∼3200 connected cell pairs (Jarrell *et al.* 2012). However, how these synapses are determined so that they are formed in reproducible patterns is still unknown. One hypothesis is the chemo-affinity hypothesis proposed by Sperry (1963), which postulates that matching pairs of cell adhesion molecules between the presynaptic and postsynaptic neurons will promote synaptogenesis. These interactions occur in the context of the surrounding extracellular matrix (ECM), the influence of which needs to be taken into account.

The ECM plays an important role in the development of the nervous system (Porcionatto 2006; Zimmermann and Dours-Zimmermann 2008; Myers *et al.* 2011). Heparan sulfate (HS) proteoglycans (HSPGs) are components of the ECM that function in several processes such as neurogenesis, cell migration, axon guidance, dendritic branching, and synapse formation (Bernfield *et al.* 1999; Bülow and Hobert 2006; Poulain and Yost 2015; Saied-Santiago and Bülow 2018). HSPGs exist in three different forms: (1) transmembrane proteins such as the syndecans; (2) glycosylphosphatidylinositol (GPI)-anchored proteins such as the glypicans; and (3) secreted forms such as perlecan, agrin, and collagen XVIII (Bernfield *et al.* 1999; Bülow and Hobert 2006; Poulain and Yost 2015). A special feature of HSPGs is the HS chains that are attached to the core proteins. These HS chains are linear glycosaminoglycan polysaccharides of variable length (*n* = 50–150) composed of hexuronic acid and glucosamine repeat units. The polysaccharide chains are substantially modified, including by deacetylation, sulfation, and epimerization. The formation of these modifications is catalyzed by specific HS modification enzymes (HSMEs) (Lindahl and Li 2009). Deacetylation is mediated by *N*-deacetylase/*N*-sulfotransferase (Ndst) enzymes, sulfation is mediated by HS 2-*O*, HS 6-*O*, HS 3-*O* sulfotransferases as well as the Ndst, and epimerization is catalyzed by the HS *C*-5 glucuronyl-epimerase. These modifications form domains of distinctly modified HSs within the HS chains that serve as binding sites for ligands and receptors. In this way, they mediate specific biological functions, such as cell migration and axon guidance (Bennett *et al.* 1997; Pratt *et al.* 2006; Matsumoto *et al.* 2007; Kastenhuber *et al.* 2009). Some of these domains may be conserved throughout evolution (Attreed *et al.* 2016).

The role of HSPGs in synapse formation and function is not well understood. In vertebrates, the HSPG agrin plays a role in the development of neuromuscular junctions by promoting the aggregation of acetylcholine receptors in skeletal muscle and activating the receptor tyrosine kinase Musk on the muscle surface (Glass *et al.* 1996). The presence of agrin has also been detected in the central nervous system, where the suppression of agrin expression in cultured hippocampal neurons and in the cortex of mice results in the formation of fewer synapses (Ferreira 1999; Bose *et al.* 2000; Ksiazek *et al.* 2007). The intracellular domains of syndecan-2 interact with various cellular components to promote filopodia formation and induce dendritic spine formation (Ethell *et al.* 2001; Lin *et al.* 2007). In *Drosophila*, the extracellular and cytoplasmic domains of syndecan act postsynaptically to regulate synapse growth of neuromuscular junctions (Nguyen *et al.* 2016). Glypicans have also been implicated in the formation of synapses. Glypican-4 (Gpc4) and glypican-6 (Gpc6) secretion from astrocytes is sufficient to induce functional synapses in retinal ganglion cells, while their elimination reduces the postsynaptic activity induced by these molecules (Allen *et al.* 2012). In addition, Gpc4 interaction with a postsynaptic leucine-rich repeat transmembrane protein (LRRTM) Lrrtm4 is required to induce excitatory synapse formation (de Wit *et al.* 2013). This interaction is mediated by an HS-dependent interaction between Gpc4 and the receptor protein tyrosine phosphatase PTPσ in the presynaptic site (Ko *et al.* 2015). Additional studies have demonstrated the involvement of HS chain modifications in the process of synapse formation. An RNAi (RNA interference) screen in *Drosophila*, specifically directed to glycan genes, revealed that the functionally paired HS 6-*O* sulfotransferase (*hs6st*) and HS 6-*O* endosulfatase (*sulf1*) have opposite effects in synaptic functional development of neuromuscular junctions (Dani *et al.* 2012). In mammals, the elimination of *Ext1*, a gene encoding an enzyme essential for HS synthesis, causes the attenuation of excitatory synaptic transmission in amygdala pyramidal neurons and results in autism-like behavioral deficits (Irie *et al.* 2012).

Here, we investigated the role of HS molecules in the development and function of the posterior male nervous system in *C. elegans*. We show that loss of the HS 3-*O* sulfotransferase HST-3.1 and the glypicans LON-2/glypican and GPN-1/glypican result in defects in response to hermaphrodite contact during male mating behavior, suggesting that 3-*O*-sulfated HS attached to LON-2/glypican and GPN-1/glypican is required for this process. In addition, HS molecules and their modifications, with the exception of 3-*O* sulfation, were required for the dorsoventral axonal migration of male-specific sensory neurons that are essential for male mating behavior and function. Loss of 3-*O* sulfation in the postsynaptic cell resulted in accumulation of a presynaptic vesicle marker in the presynaptic cell of a mating circuit. Further, synapse formation between male-specific sensory neurons and target interneurons was disrupted, possibly accounting for the observed behavioral defect.

## Materials and Methods

### *C. elegans* strains and imaging

All strains were maintained using standard methods (Brenner 1974). All strains used contain the *him-5(e1490)* mutation on chromosome V to increase the male population (Broverman 1994). We refer to *him-5* as control worms. All experiments were performed at 20°, and animals were scored as 1-day-old adults unless otherwise specified. The strains and mutant alleles used in this study are listed in the supplemental experimental procedures. Fluorescent images were captured in live *C. elegans* using a Plan-Apochromat 40×/1.4 or 63×/1.4 objective on a Zeiss Axioimager Z1 Apotome (Zeiss [Carl Zeiss], Thornwood, NY). Worms were immobilized using 10 mM sodium azide and z-stacks were collected. Maximum intensity projections were used for further analysis.

### Molecular biology and transgenesis

To assemble tissue-specific expression constructs used for rescue experiments, the *hst-3.1* cDNA was cloned under control of the following promoters: hypodermal *Pdpy-7* (Gilleard *et al.* 1997), body wall muscle *Pmyo-3* (Okkema *et al.* 1993), pan-neuronal *Prgef-1* (Altun-Gultekin *et al.* 2001), the B-type ray neurons *Ppkd-2* (Barr and Sternberg 1999), EF interneurons *Pnlg-1* (this study), dopaminergic neurons *Pcat-2* (Lints and Emmons 1999), PVY and PVX neurons *Pnlp-14* (Sherlekar *et al.* 2013), AVA neuron *Pnmr-1* (Sherlekar *et al.* 2013), glutamatergic neurons *Peat-4* (Lee *et al.* 1999), serotonergic neurons *Ptph-1* (Sze *et al.* 2000), and γ-aminobutyric acid (GABA)ergic neurons *Punc-47* (Gendrel *et al.* 2016). All plasmids contained the *unc-54* 3′-UTR. Constructs for tissue-specific rescue experiments of the *hst-3.1/*HS 3-*O*-sulfotransferase male mating response defect were injected at 5 ng/µl together with *Pceh-22*::*GFP* or *Punc-122*::*GFP* as injection markers at 50 ng/µl. For details see the supplemental experimental procedures.

### Behavioral scoring

Response to hermaphrodite contact assays were performed with 1-day-old adult, virgin males isolated at the L4 stage. Male worms to be tested were placed on a 10-mm food lawn with ten *unc-31(e169)* hermaphrodites. The mating behavior of the males was observed for 5 min and annotations were made every time a male responded to hermaphrodite contact. For this assay, a male is considered to have responded to contact if, after mate contact, it starts backward locomotion, scanning the hermaphrodite body followed by turning behavior. Males that failed to respond to contact did not start backward locomotion to scan the hermaphrodite body, or they lost tail contact right after starting the backward locomotion. The quantitation of response to contact was performed by dividing the number of males with response by the total number of male tested [response % = (number of males with response / total number of males evaluated) × 100. For statistical analysis, we performed a Student’s *t*-test to calculate the significant difference between control worms (*him-5*) and mutant worms (in a *him-5* background).

For the mating potency assay, we placed one virgin young adult male worm with one *pha-1* mutant hermaphrodite on a 10-mm food lawn for 4 hr. We scored a total of 50 male worms (50 plates). After 4 hr, males were removed from the plate and the plate was placed at 25°. The *pha-1* mutant worms are temperature-sensitive and are not viable at 25°, so only the crossed progeny grows at 25°. Three days later, we counted the plates with worms that survived at 25°. To calculate mating potency, we divided the number of cross progeny plates by the total number of plates/males tested [mating potency % = (number of plates/males with cross progeny / total number of plates/males tested) × 100]. For statistical analysis, we performed a Student’s *t*-test to calculate the significant difference of mating potency between wild-type worms (*him-5*) and mutant worms (in a *him-5* background).

For backing response after nose touch, we placed one virgin young adult male worm in a clean bacterial lawn and gently touched its nose 10 times with an eyelash, waiting 10 sec between each touch. The number of times the worm showed response to nose touch by backward movement was scored and the backing response was calculated [backing response = (number of times the worm backed up after nose touch / 10 nose touches) × 100]. For statistical analysis, we performed a Student’s *t*-test to calculate the significant difference of backing response between wild-type worms (*him-5*) and mutant worms (in a *him-5* background).

### RnB synapse imaging

To visualize the presynaptic pattern of RnB neurons, z-stack images of young adult males containing *Ppkd-2*::*GFP* and *Ppkd-2*::*mCherry*::*RAB-3* reporters (*bxIs30*) were acquired using a Leica SP5 confocal microscope. The z-stack images were analyzed one by one from ventral to dorsal and compressed by looking at the synaptic puncta located in the preanal ganglion. To quantify the protein levels of *mCherry*::*RAB-3*, we performed a fluorescent densitometry analysis of compressed z-stacks images acquired by using a Zeiss Axioimager Z1 Apotome with a 63×/1.4 objective. For this analysis, we used the same exposure time for all control and mutant samples. The relative fluorescence values were measured by dividing *mCherry* densitometry (corresponding to synapses) by *GFP* densitometry (corresponding to axon terminals). In this way, we corrected for missing synapses that are a product of defects in axonal migration. For statistical analysis, we performed a Student’s *t*-test to calculate the significant difference between control worms and mutant worms.

To visualize the synapses between RnB ray sensory neurons and EF interneurons, we used the iBLINC (Biotin Labeling of INtracellular Contacts) *trans*-synaptic biotin transfer system (Desbois et al., 2015). For the presynaptic RnB sensory neuron labeling, we expressed the biotin ligase with *nrx-1* fusion protein (*BirA*::*NRX-1*) driven by the *pkd-2/*polycystin-2 promoter. For the postsynaptic EF interneuron labeling, we expressed the biotinylated acceptor peptide with an *nlg-1*/neuroligin fusion protein (*AP*::*NLG-1*) driven by the *nlg-1*/neuroligin promoter. We coexpressed these pre- and postsynaptic fusion proteins with *streptavidin*::*RFP* (red fluorescence protein) fusion protein driven by the *unc-122* coelomocyte promoter. To quantify RnBs → EF biotinylated synapses, we performed a fluorescent densitometry analysis of compressed z-stack images acquired from mutant and wild-type worms by using a Zeiss Axioimager Z1 Apotome with a 63×/1.4 objective. Only those worms completely oriented in a dorsoventral position were imaged to obtain a clear view of the synaptic ring located in the preanal ganglion. The relative fluorescent [arbitrary unit (a.u.)] values were the measurement of the *RFP* densitometry subtracted by the background (noise). The measured area consists of a circle with a 5-μm radius. To compare the difference in synaptic densities between mutant and wild-type worms, we divided the relative fluorescence (a.u.) value of mutants by the average of the relative fluorescence (a.u.) of wild-type worms measured on the same experimental day. For statistical analysis, we performed a Student’s *t*-test to calculate the significant difference between wild-type worms and mutant worms.

### Data availability

All strains and reagents are available upon request. Supplemental Material, File S1, File S2, and File S3 comprise all data used to create the figures. File S4 contains supplemental figures and tables. Figure S1 in File S4 shows the analysis of male potency, response defects, and general backing response in the *hst-3.1/*HS 3-*O*-sulfotransferase. Figure S2 in File S4 provides additional genetic data pertaining to the genetic interactions between *hst-3.1/HS 3-O sulfotransferase*, *pkd-2/*polycystin-2, *lov-1/*polycystin-1, and *klp-6/*kinesin. Figure S3 in File S4 shows expression data of the *hst-3.1/*HS 3-*O*-sulfotransferase transcriptional reporter and *nlg-1*/neuroligin transcriptional GFP fusion. Figure S4 in File S4 provides additional genetic data pertaining to the role of HSMEs and HSPGs in the axon guidance of B-type ray neurons. Figure S5 in File S4 provides additional information about the visualization of the B-type ray neuron synapses with the EF interneurons. Figure S6 in File S4 provides additional genetic data pertaining to the quantification of *mCherry*::*RAB-3* in the HSME and HSPG mutants. Table S1 in File S4 provides a complete list of strains created for this study. Tables S2 and S3 in File S4 provide a complete list of transgenic strains, and the respective constructs, created for this study.

## Results

### Genetic elimination of HS modification enzymes and HSPGs causes behavioral defects in male mating

**Figure 1:**
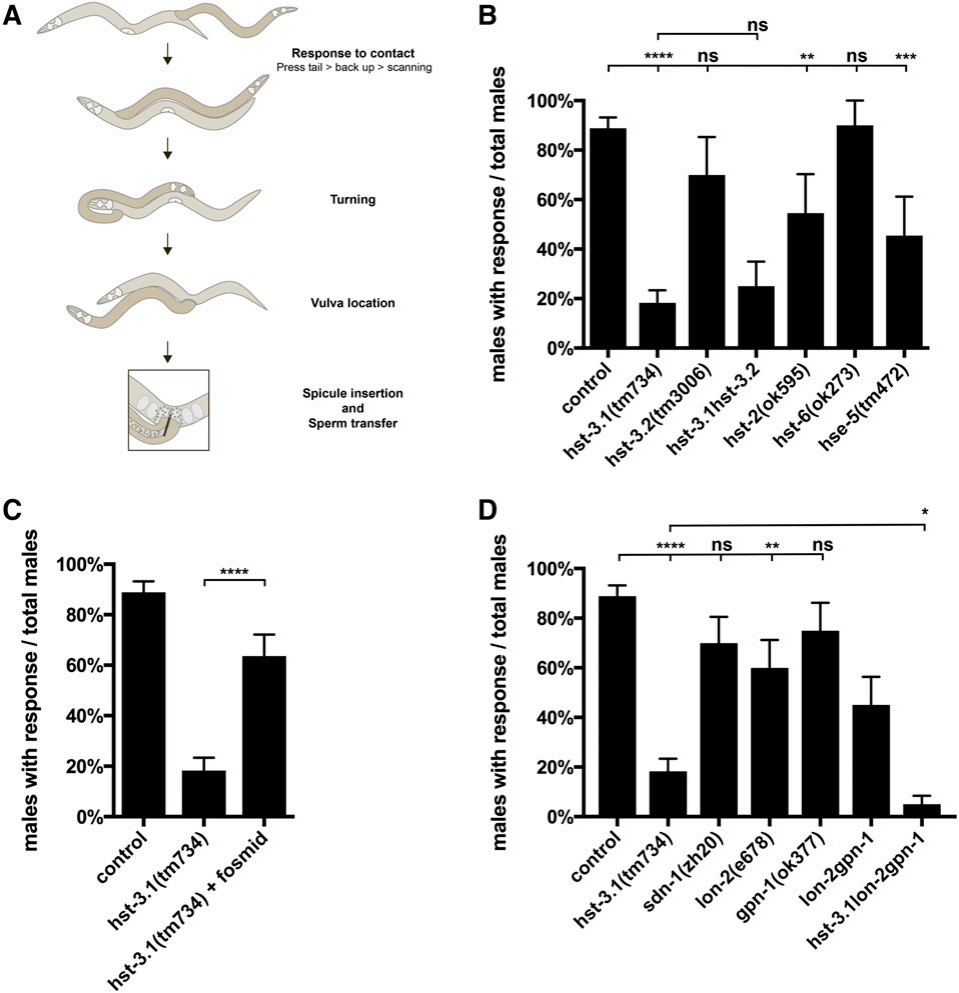
Heparan sulfate modification enzymes and heparan sulfate proteoglycans are required for the response to hermaphrodite contact during male mating behavior. (A) Schematic of the steps of male mating behavior. (B and D) Quantification of the response to hermaphrodite contact during male mating behavior in the genotypes indicated. Error bars denote the SEM; statistical significance is shown as follows: * *P* < 0.05, ** *P* < 0.005, *** *P* < 0.0005, and **** *P* < 0.00005. ns, not significant. The data for control are identical (B–D) and shown for comparison only. (C) Quantification of a *hst-3.1*-containing fosmid rescue of the response to hermaphrodite contact during male mating behavior in the *hst-3.1(tm734)* mutant. Error bars denote the SEM; statistical significance is shown as follows: * *P* < 0.05, ** *P* < 0.005, *** *P* < 0.0005, and **** *P* < 0.00005. ns, not significant. The data for control and *hst-3.1* are identical to (B) and shown for comparison only.

Behavior provides a sensitive readout of developmental or functional disruptions. We conducted a response to hermaphrodite contact behavioral assay of male worms carrying null mutations for the HS modification enzymes. Response to hermaphrodite contact is the decision of a male worm to start mating after contact by pressing its tail against the hermaphrodite body while moving backward searching for the vulva (Figure 1A). We observed that males carrying a single mutation in *hst-2/*HS 2-*O*-sulfotransferase, *hse-5/*HS *C*-5-epimerase, and *hst-3.1/*HS 3-*O*-sulfotransferase showed deficiency in response to contact. Loss of *hst-2/*HS 2-*O*-sulfotransferase or *hse-5/*HS *C*-5-epimerase, which introduce 2-*O*-sulfation and *C*-5 epimerization in the hexuronic acid of the linear glycosaminoglycan HS chains, respectively, mildly affected the response to hermaphrodite contact. Forty-five percent of *hst-2(ok595)* and 55% of *hse-5(tm472)* mutant males failed to respond after tail contact compared to a 10% failed response in wild-type worms (Figure 1B). For mutants in *hst-3.1(tm734)*, which introduces 3-*O*-sulfation, 82% of the *hst-3.1(tm734)* mutant males failed to respond (Figure 1B). This reduction in response to contact results in a reduced number of cross progeny (Figure S1A in File S4), demonstrating that the *hst-3.1(tm734)* mutant defect in response reduced the overall ability to succeed in mating. Both response and mating potency defects were rescued by a fosmid containing the *hst-3.1* locus (Figure 1C and Figure S1A in File S4). The *hst-3.1(tm734)* mutant deficiencies in response behavior involved a failure in backward movement after mate contact; all nonresponsive mutant worms either failed to back-up after hermaphrodite contact (52%) or showed a discontinued backward movement losing contact with the hermaphrodite (30%) (Figure S1B in File S4). This defect in backward locomotion is not caused by a general defect in backing behavior as *hst-3.1(tm734)* mutant males did not show defects in the backing response to nose touch (Figure S1C in File S4). However, since *hst-3.1(tm734)* mutant worms showed severe defects in response to contact, subsequent male mating behavior steps were not examined.

The abnormal response to mate contact due to loss of 3-*O*-sulation was specific for *hst-3.1/*HS 3-*O*-sulfotransferase, since male worms of *hst-3.2(tm3006)*, a null allele of the other 3-*O*-sulfatransferase, did not show defects in response after tail contact during mating (Figure 1B). A double mutant for *hst-3.1* and *hst-3.2* did not enhance the abnormal response phenotype of *hst-3.1(tm734)* single mutants, suggesting that 3-*O*-sulfation by *hst-3.1/*HS 3-*O*-sulfotransferase, but not *hst-3.2/*HS 3-*O*-sulfotransferase, is required to mediate male mating behavior. On the other hand, *hst-6/*HS 6-*O*-sulfotransferase, which introduces 6-*O*-sulfation, does not serve an individual role in response to hermaphrodite contact as 90% of *hst-6(ok273)* mutant males showed a response after tail contact. Taken together, these results indicate that HS molecules modified by *hst-3.1/*HS 3-*O*-sulfotransferase, *hst-2/*HS 2-*O*-sulfotransferase, and *hse-5/*HS *C*-5-epimerase, but not *hst-6/*HS 6-*O*-sulfotransferase, are required for response to hermaphrodite contact by the male worm.

Since *hst-3.1* is a 3-*O*-sulfotransferase that modifies the HS chains on HSPGs, we wanted to determine which HSPG may contain the epitope with 3-*O* sulfation that is required for response to hermaphrodite contact. We tested mutants of *sdn-1*/syndecan and the two forms of glypicans in the worm, *lon-2*/glypican and *gpn-1*/glypican. The *sdn-1(zh20)* mutant worms were not defective in response to hermaphrodite contact (Figure 1D). Single-mutant worms for *lon-2(e678)* were significantly different from control worms as 60% showed a response to contact compared to ∼90% for control males. This *lon-2* defect is not due to the anatomical Lon phenotype as *lon-1(e185)* mutant males, which have an elongated body similar to *lon-2*, did not show defects in response to hermaphrodite contact (Figure S1D in File S4). *gpn-1(ok377)* males were not significantly different from control worms in their response to hermaphrodites. However, the *lon-2(e678) gpn-1(ok377)* double-mutant worms further enhanced the defect observed in the *lon-2* single mutants, indicating that *gpn-1* also has a function that promotes response (Figure 1D).

To further determine if *hst-3.1/*HS 3-*O*-sulfotransferase is acting on the HS chains attached to *lon-2*/glypican and *gpn-1*/glypican proteoglycans to regulate response behavior, we constructed triple mutants for *hst-3.1/*HS 3-*O*-sulfotransferase, *lon-2*/glypican, and *gpn-1*/glypican. The triple-mutant defect was enhanced as compared to the *hst-3.1/*HS 3-*O*-sulfotransferase single mutant (Figure 1D), suggesting that LON-2/glypican and GPN-1/glypicans have further functions in promoting response independent of 3-*O*-sulfation by *hst-3.1*.

### HS 3-*O* sulfation in the EF interneurons regulates male response to hermaphrodite contact

**Figure 2:**
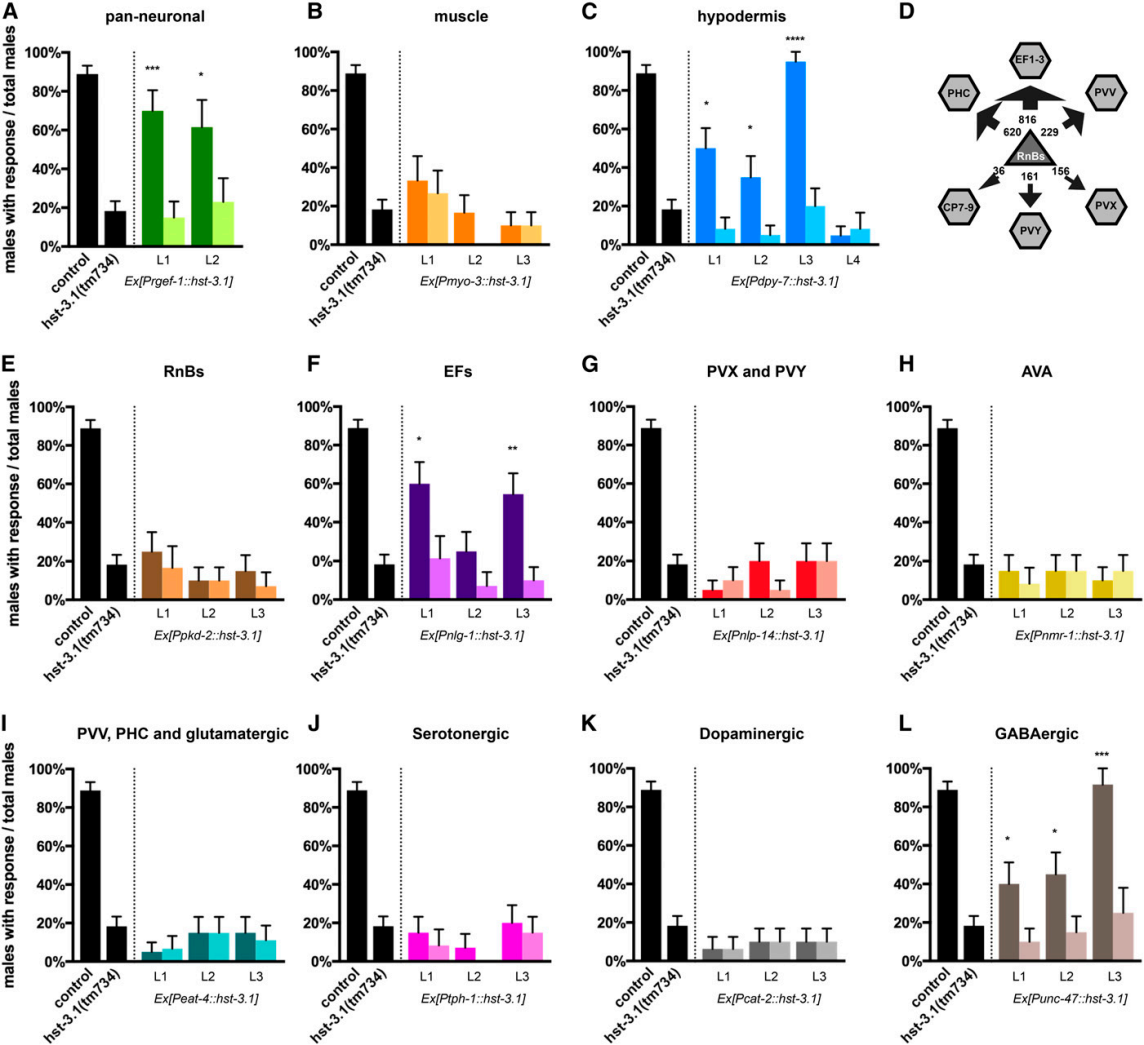
Heterologous transgenic rescue experiments. (A–C) Tissue-specific rescue of response to hermaphrodite contact during male mating behavior in *hst-3.1(tm734)* mutants with *hst-3.1* cDNA under heterologous promoters as indicated. Rescue was defined as restoration of response to hermaphrodite contact during male mating in transgenic animals (darker shade) and had to be statistically significant (*P* < 0.05) compared to nontransgenic siblings (lighter shade) (*n* ≥ 12). (D) Main postsynaptic partners of RnB neurons. The arrows and numbers represent the weight of the synaptic input from RnBs to the other neurons. (E–L) Cell-specific rescue of response to hermaphrodite contact during male mating behavior in *hst-3.1(tm734)* mutants with *hst-3.1* cDNA under heterologous promoters as indicated. Rescue was defined as restoration of response to hermaphrodite contact during male mating in transgenic animals (darker shade) and had to be statistically significant (*P* < 0.05) compared to nontransgenic siblings (lighter shade) (*n* ≥ 12). GABA, γ-aminobutyric acid.

To define the focus of action of the *hst-3.1/*HS 3-*O*-sulfotransferase in its role in response to contact during mating, we expressed the *hst-3.1/*HS 3-*O*-sulfotransferase cDNA tissue-specifically in neurons, muscles, and the hypodermis. When we expressed *hst-3.1/*HS 3-*O*-sulfotransferase in neurons using the *rgef-1* pan-neuronal promoter (*_p_rgef-1*::*hst-3.1*), we observed rescue of the abnormal response to contact phenotype (Figure 2, A–C). Similarly, when we expressed a *hst-3.1/*HS 3-*O*-sulfotransferase cDNA in hypodermal tissue using the *dpy-7* promoter (*_p_dpy-7*::*hst-3.1*), we observed rescue of the mutant phenotype. However, when we expressed *hst-3.1/*HS 3-*O*-sulfotransferase in muscles using the *myo-3* promoter (*_p_myo-3*::*hst-3.1*), we were not able to rescue the response defect. These results suggest that the expression of *hst-3.1/*HS 3-*O*-sulfotransferase in neurons or the hypodermis, but not in muscles, is sufficient to regulate response behavior in the male worms.

To further delineate the neuronal focus of action, we used cell-specific promoters that drove expression of the *hst-3.1/*HS 3-*O*-sulfotransferase cDNA in subsets of neurons in the male tale. Cell ablation experiments indicated that the B-type ray sensory neurons are essential for male mating behavior, particularly for the response to hermaphrodite contact and vulva location steps (Liu and Sternberg 1995; Barr and Sternberg 1999; Koo *et al.* 2011). By examining double mutants, we found that genes such as *pkd-2/*polycystin-2, *lov-1/*polycystin-1, and *klp-6*/kinesin, which act cell-autonomously in RnB neurons to mediate the response to hermaphrodite contact (Barr *et al.* 2001; Peden and Barr 2005), act genetically in the same genetic pathway as *hst-3.1/*HS 3-*O*-sulfotransferase (Figure S2 in File S4). However, expression of *hst-3.1/*HS 3-*O*-sulfotransferase in B-type ray neurons by using the *pkd-2/*polycystin-2 cell-specific promoter (*_p_pkd-2*::*hst-3.1*) was not sufficient to rescue the abnormal response phenotype (Figure 2E). Therefore, to determine whether *hst-3.1/*HS 3-*O*-sulfotransferase is acting downstream of the B-type sensory neurons to regulate response, we expressed the *hst-3.1/*HS 3-*O*-sulfotransferase cDNA in their main postsynaptic partners. Based on the EM male connectivity data, the main postsynaptic targets of RnB neurons are the EF_(1–3)_, PVX, PVY, PVV, PHC, and CP_(7–8)_ male-specific interneurons (Jarrell *et al.* 2012) (Figure 2D). In addition, PVX and PVY are heavily connected to the AVA command interneuron, and previous studies revealed that these three interneurons are essential for the backup locomotion that triggers response behavior after mate contact (Sherlekar *et al.* 2013). To express *hst-3.1/*HS 3-*O*-sulfotransferase in the EF_(1–3)_ interneurons, we used an *nlg*-1/neuroligin promoter sequence (*_p_nlg-1*::*hst-3.1*), since expression of *nlg-1* in EF_(1–3)_ interneurons has been observed by using a transcriptional GFP fusion (Figure S3C in File S4). Expression of *hst-3.1/*HS 3-*O*-sulfotransferase in the EF_(1–3)_ interneurons was sufficient to rescue the abnormal phenotype in response, as ∼60% of the male worms responded well after tail contact compared to 20% of response in nontransgenic siblings. On the other hand, expression of *hst-3.1/*HS 3-*O*-sulfotransferase cDNA in PVX, PVY, PVV, PHC, and AVA interneurons was not sufficient to rescue the abnormal response phenotype in mating behavior (Figure 2, F–I). A previously published transcriptional reporter for *hst-3.1/*HS 3-*O*-sulfotransferase (Tecle *et al.* 2013) is not expressed in EF interneurons (or the hypodermis) and, consistent with this observation, expression of the *hst-3.1/*HS 3-*O*-sulfotransferase cDNA under the same promoter fails to rescue the male mating defects in *hst-3.1/*HS 3-*O*-sulfotransferase mutant males (Figure S3 in File S4). However, it only contains ∼3 kb of sequences upstream of the starting codon and therefore may be missing key regulatory sequences for proper expression of *hst-3.1*. For this reason, we cannot discard the possibility that *hst-3.1/* HS 3-*O*-sulfotransferase might be expressed in the EF_(1–3)_. Altogether, based on the heterologous expression of *hst-3.1/*HS 3-*O*-sulfotransferase, we suggest that *hst-3.1/*HS 3-*O*-sulfotransferase acts in the EF_(1–3)_ male-specific interneurons, which are important postsynaptic targets of B-type ray neurons, to regulate response behavior during male mating.

Additionally, we tested whether *hst-3.1/*HS 3-*O*-sulfotransferase could act in serotonergic, dopaminergic, glutamatergic, and GABAergic neurons by expressing it with the *tph-1* promoter (*_p_tph-1*::*hst-3.1*), *cat-2* promoter (*_p_cat-2*::*hst-3.1*), *eat-4* promoter (*_p_eat-4*::*hst-3.1*), or unc-47 promoter (*_p_unc-47*::*hst-3.1*), respectively. Expression of *hst-3.1* cDNA in serotonergic, dopaminergic, and glutamatergic neurons did not rescue the abnormal response phenotype (Figure 2, I–K). However, expression of *hst-3.1* cDNA in GABAergic neurons rescued the response to hermaphrodite contact defects (Figure 2L). This is consistent with rescue in GABAergic EF interneurons (Gendrel *et al.* 2016).

### HS 3-*O* sulfation is not required for axon guidance of the B-type ray sensory neurons

Since HS molecules are mediators of axon guidance in many neurons in *C. elegans* (Bülow and Hobert 2004; Kinnunen *et al.* 2005; Bülow *et al.* 2008; Kinnunen 2014), we wanted to determine whether the observed behavioral defects in *hst-3.1/*HS 3-*O*-sulfotransferase mutant worms were the result of guidance defects of neurons involved in response behavior. As previously mentioned, the B-type ray sensory neurons are mediators of response to contact behavior and our findings suggest that *hst-3.1/*HS 3-*O*-sulfotransferase is acting from their main postsynaptic target, the EF interneurons, to mediate this behavior. To determine whether 3-*O* sulfation by HST-3.1*/*HS 3-*O*-sulfotransferase in the EF interneurons is acting as a guidance cue to regulate axon migration, we looked at B-type ray neuron axonal processes in *hst-3.1/*HS 3-*O*-sulfotransferase mutant worms. To visualize B-type ray neuron processes, we used a cytoplasmic *GFP* reporter driven by the *pkd-2/*polycystin-2 promoter. *pkd-2/*polycystin-2 encodes a transient receptor potential polycystic (TRPP) cation channel that is expressed in three types of male-specific sensory neurons: the RnBs, HOB, and the CEMs.

**Figure 3:**
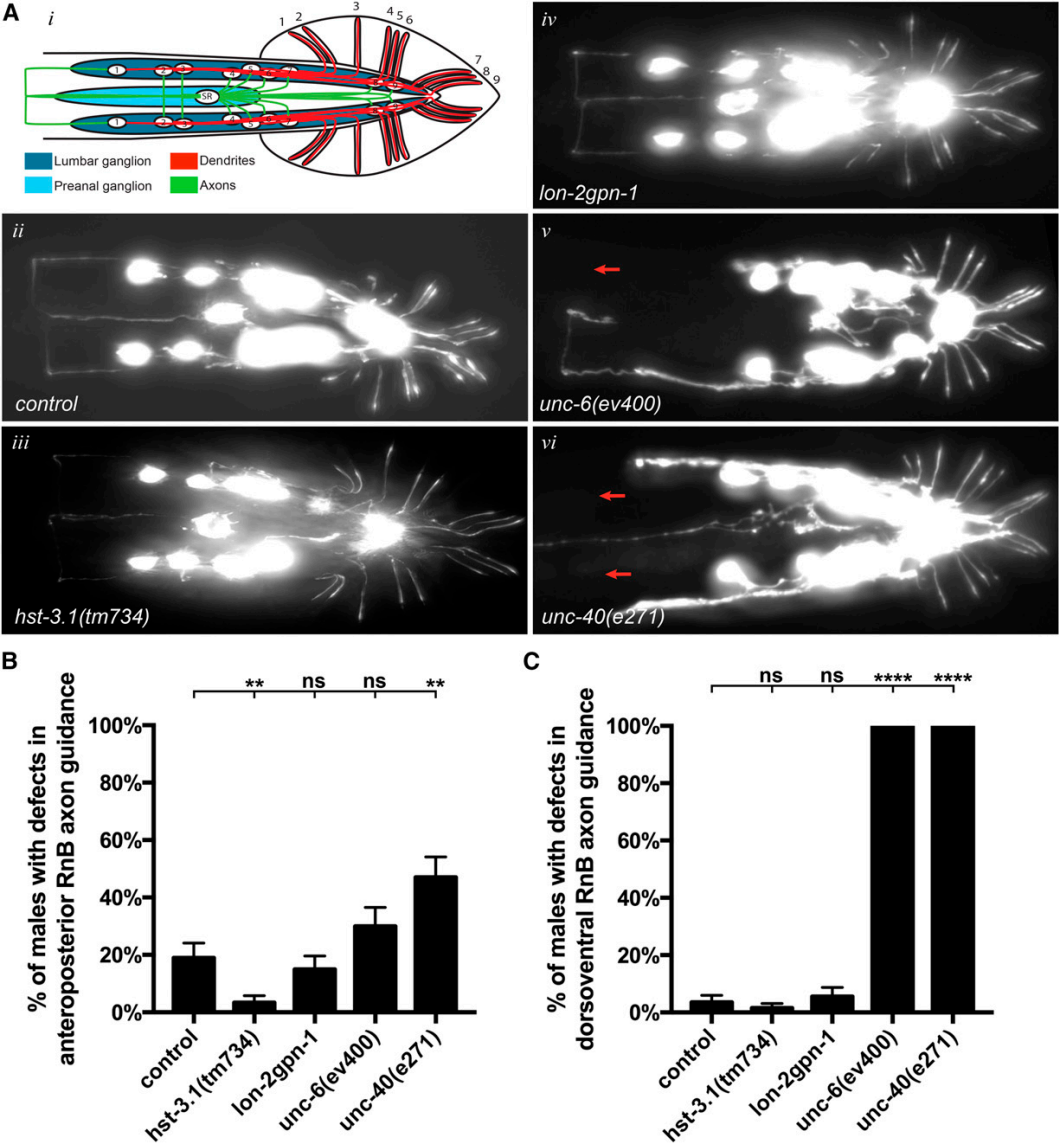
HST-3.1*/*HS 3-*O*-sulfotransferase is not required for axon guidance of B-type ray neurons. (A) Ventral views with schematics (*i–vi*) of adult male animals showing the B-type ray neurons. B-type ray neurons were visualized with *bxIs14* (*Is*[*Ppkd-2*::*GFP*]). Red arrows represent missing commissures. Anterior is to the left. (B and C) Quantification of B-type ray neurons anteroposterior and dorsoventral axon guidance in the genotypes indicated. Error bars denote the SEM; statistical significance is shown as follows: * *P* < 0.05, ** *P* < 0.005, *** *P* < 0.0005, and **** *P* < 0.00005. ns, not significant.

In wild-type worms, during the mid- to late-L4 stage, ray neuron cell bodies migrate away from the posterior tail hypodermis and enter the lumbar ganglion (Sulston *et al.* 1980) (Figure S4A in File S4). The axons migrate out of the lumbar ganglion in a dorsoventral (DV) pathway, forming four to five circumferential commissures. On the ventral side, the axon terminals enter the preanal ganglion where they contact their postsynaptic partners. In the case of R1B and sometimes R2B, the axons first migrate in an anteroposterior (AP) pathway, then changing to the DV migration. Therefore, defects in AP migration of B-type ray neurons result in an anterior overextension of processes, while defects in DV migration result in the absence of commissures. As previously reported (Jia and Emmons 2006), these migratory defects are observed in mutant worms for the netrin signaling pathway, where *unc-6(ev400)* and *unc-40(e271)* single mutants showed 30 and 47%, respectively, of defects in AP migration, while both mutants showed 100% of defects in DV migration (Figure 3). Interestingly, other HS modifications such as 2-*O* sulfation, 6-*O* sulfation, and 5-*C* epimerization mediate B-type ray neuron axon guidance, as defects are observed in both AP and DV migratory pathways of *hst-2(ok595)*, *hst-6(ok273)*, and *hse-5(tm472)* single-mutant worms (Figure S4, C and D in File S4). In addition, SDN-1 is a regulator of B-type ray neuron axon guidance as defects in both AP and DV migration were observed in *sdn-1(zh20)* single mutants. The *hst-6(ok273) hst-2(ok595)* and *lon-2(e678) sdn-1(zh20)* double mutants showed highly penetrant AP and DV migration defects. In fact, the DV migration defects of these double mutants are comparable to those observed in netrin signaling mutants, suggesting that HS molecules and their distinct HS modification patterns regulate B-type ray neuron axon guidance through the *unc-6*/netrin ligand system (Figure 3 and Figure S4, C–F in File S4).

In contrast, the axon morphology of B-type ray neurons in *hst-3.1(tm734)* single-mutant worms was indistinguishable from control worms, with no defects observed in the AP or DV axon migration of RnB neurons (Figure 3). The same results were obtained when we examined the *lon-2gpn-1* double mutants, which are also defective for response behavior, where no defects in RnB neuron AP or DV axon migration were observed. These results suggest that the defects in response to hermaphrodite behavior in *hst-3.1/*HS 3-*O*-sulfotransferase and *glypican* mutants are not a consequence of defective axonal projections of B-type ray neuron function. Taken together, our analysis of the roles of HS molecules in axon guidance suggests that specific HS modifications are required for axonal migration of B-type ray neurons, whereas, 3-*O* sulfation by HST-3.1/HS 3-*O*-sulfotransferase is not required for this process but may rather serve different functions.

### HS 3-*O* sulfation mediates synapse formation of B-type ray sensory neurons

**Figure 4:**
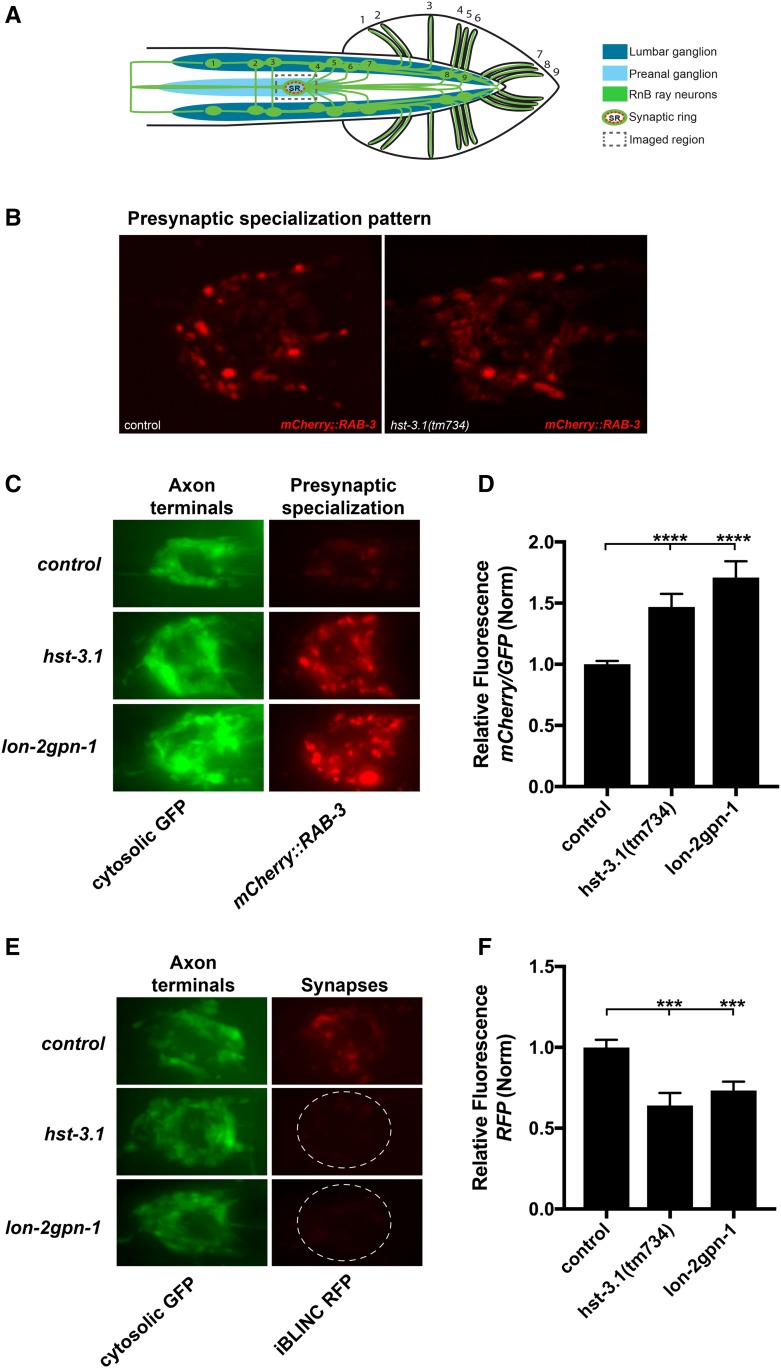
HST-3.1*/*HS 3-*O*-sulfotransferase regulates presynaptic organization and synapse formation of B-type ray neurons. (A) Schematic of a ventral view showing the imaged region containing the synaptic ring in the preanal ganglion. (B) Confocal ventral views of the presynaptic densities as labeled with mCherry::RAB-3 of adult male animals in control and *hst-3.1* mutant. The B-type ray neuron presynaptic distribution is not affected in *hst-3.1/*HS 3-*O*-sulfotransferase mutants. (C) Ventral views of the RnB axonal processes in the synaptic ring located in the preanal ganglion with cytosolic GFP and its corresponding presynaptic densities as labeled with mCherry::RAB-3 of adult *hst-3.1(tm734)* single mutants and *lon-2gpn-1* double-mutant male worms. B-type ray neurons were visualized with *bxIs30* that contains the cytosolic GFP (*Is*[*Ppkd-2*::*GFP*]) and the presynaptic marker (*Is*[*Ppkd-2*::*mCherry*::*RAB-3*]). Anterior is to the left. (D) Quantification of mCherry::RAB-3 fluorescence in the preanal ganglion synaptic ring in the genotypes indicated. Error bars denote the SEM; statistical significance is shown as follows: * *P* < 0.05, ** *P* < 0.005, *** *P* < 0.0005, and **** *P* < 0.00005. ns, not significant. The data presented is a ratio of mCherry::RAB-3 to GFP and control value. (E) Ventral views of *trans*-synaptic biotinylation labeling (iBLINC) of RnB → EFs synapses in puncta in *hst-3.1(tm734)* single mutants and *lon-2gpn-1* double mutants. (F) The data presented is the normalized red fluorescence protein density in the synaptic area. Error bars denote the SEM; statistical significance is shown as follows: * *P* < 0.05, ** *P* < 0.005, *** *P* < 0.0005, and **** *P* < 0.00005. ns, not significant.

The observation that *hst-3.1/*HS 3-*O*-sulfotransferase mutants are defective in response to contact behavior during male mating, but do not display any obvious defects in axonal projections, prompted us to investigate whether HS 3-*O* sulfation is regulating the synaptic function of the response circuits. To examine the possibility that *hst-3.1/*HS 3-*O*-sulfotransferase is affecting synapse formation of B-type ray neurons, we investigated the presynaptic densities of mutant worms using the *mCherry*::*RAB-3* reporter expressed under the control of the *pkd-2/*polycystin-2 promoter. *rab-3* encodes a member of the Ras GTPase superfamily that localizes to presynaptic vesicles. To study RnB synapses, we used a confocal microscope and analyzed the synaptic pattern of compressed z-stacks containing all the RnB synapses located in the preanal ganglion. We looked at the synapses of young adult male worms (64 hr after hatching) because by this time most B-type ray neuron synapses are formed (Figure S5A in File S4). Based on the presynaptic puncta distribution in the preanal ganglion, the presynaptic density patterning of *hst-3.1/*HS 3-*O*-sulfotransferase mutant males was similar in morphology to that of control worms, suggesting that the elimination of *hst-3.1/*HS 3-*O*-sulfotransferase does not affect the distribution of B-type ray neuron presynaptic sites (Figure 4B). However, we found that the *mCherry*::*RAB-3* levels are accumulated above control levels (Figure 4C).

To quantify the levels of *mCherry*::*RAB-3* in synapses, we performed a densitometry analysis of compressed z-stack images containing all RnB synapses in the preanal ganglion. For this analysis, we used the same exposure times for mutants and control worms. To control for transgene variability, we normalized by using a cytoplasmic GFP expressed from the same transgene and promoter. We observed that *hst-3.1/*HS 3-*O*-sulfotransferase mutants have a *mCherry* relative fluorescence of 1.47 ± 0.11 compared to 1 ± 0.03 in control worms (Figure 4D).

The *mCherry*::*RAB-3* levels in the B-type neuron synapses of *lon-2gpn-1* double-mutant worms have a similar accumulation of RAB-3, with a relative fluorescence of 1.71 ± 0.13 compared to 1 ± 0.03 in control worms (Figure 4D). The *lon-2(e678)* single-mutant worms, which are defective for response to contact, also showed increased levels of RAB-3 in B-type ray neuron synapses when compared to control (Figure 1D and Figure S6B in File S4).

Interestingly, mutant worms for the other HSME with defects in male mating behavior, *hse-5(tm472)*, also showed higher *mCherry*::*RAB-3* accumulation levels significantly different to those of control worms (Figure 1B and Figure S6A in File S4). However, given that these mutants are also defective in axon guidance (Figure S4, C and D in File S4), the observed behavioral defects might be due to axon misrouting rather than synaptic function, as seems to be the case for *hst-3.1/*HS 3-*O*-sulfotransferase. Finally, *hst-3.2(tm3006)* and *hst-6(ok273)* mutant worms did not show defects in the *mCherry*::*RAB-3* accumulation in synapses, nor did they show a defect in response to contact behavior (Figure 1B and Figure S6A in File S4). The single and double HSPG mutants such as *sdn-1(zh20)*, *gpn-1(ok377)*, *unc-52(e998)*, *lon-2sdn-1*, and *sdn-1gpn-1*, did not show different levels of accumulation of *mCherry*::*RAB-3* in presynaptic sites compared to control worms (Figure S6B in File S4).

The abnormal *mCherry*::*RAB-3* accumulation observed in B-type ray neurons of *hst-3.1/*HS 3-*O*-sulfotransferase and *lon-2gpn-1* mutant worms suggests a synaptic disruption between these neurons and their postsynaptic partners. To examine this possibility, we used the iBLINC system to label the specific synapses between B-type ray neurons and EF interneurons. The iBLINC method consists of an enzymatic *trans*-synaptic transfer reaction of biotin from a presynaptic cell adhesion protein to a postsynaptic molecule (Desbois *et al.* 2015). The biotinylated postsynaptic site is detected by streptavidin::fluorescent protein, thereby labeling the synapse (Figure S5B in File S4). For the B-type ray neurons, we expressed a BirA ligase fused N-terminally to NRX-1/neurexin driven by the *pkd-2/*polycystin-2 promoter. For the EF interneurons, we expressed an acceptor peptide AP fused N-terminally to NLG-1/neuroligin driven by the *nlg-1*/neuroligin promoter. Using iBLINC, we found that *hst-3.1(tm734)* mutants show less synaptic labeling compared to control worms, suggesting smaller or fewer synapses between RnBs and the EF neurons. Control worms have a relative fluorescence of 1 ± 0.05, while the *hst-3.1(tm734)* mutants have a relative fluoresce of 0.26 ± 0.03 (Figure 4, E and F). Moreover, *lon-2gpn-1* double-mutant worms also showed less synaptic labeling than control worms with a relative fluorescence of 0.75 ± 0.05. Altogether, our synaptic and behavioral results suggest that HS 3-*O* sulfation is involved in the process of synapse formation, which in turn may affect the synaptic function of the connection between B-type ray neurons and EF interneurons, resulting in the defects in response behavior during male mating.

### HST-3.1*/*HS 3-*O*-sulfotransferase and the glypicans LON-2/glypican and GPN-1/glypican genetically interact with synaptic molecules

**Figure 5:**
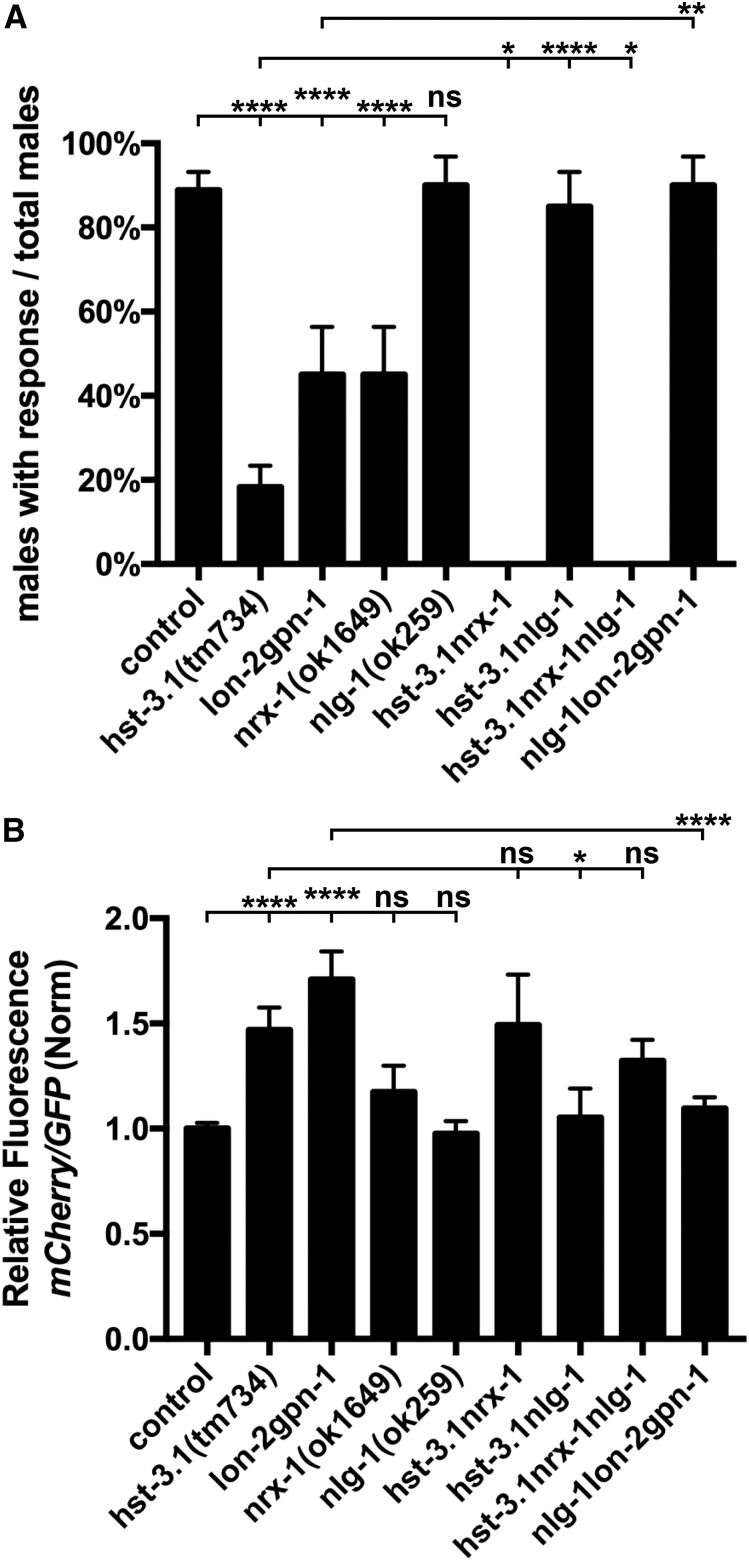
*nrx-1*/neurexin and *nlg-1*/neuroligin interacts genetically with *hst-3.1/*HS 3-*O*-sulfotransferase for response to hermaphrodite contact and synaptic function. (A) Quantification of response to hermaphrodite contact during male mating behavior in the genotypes indicated. Error bars denote the SEM; statistical significance is shown as follows: * *P* < 0.05, ** *P* < 0.005, *** *P* < 0.0005, and **** *P* < 0.00005. ns, not significant. The data for control and *hst-3.1/* HS 3-*O*-sulfotransferase are identical to Figure 1 and shown for comparison only. (B) Quantification of mCherry::RAB-3 fluorescence in the preanal ganglion synaptic ring in the genotypes indicated. The data presented is a ratio of mCherry::RAB-3 to GFP and control (Norm) value. Error bars denote the SEM; statistical significance is shown as follows: * *P* < 0.05, ** *P* < 0.005, *** *P* < 0.0005, and **** *P* < 0.00005. ns, not significant. The data for control and *hst-3.1/*HS 3-*O*-sulfotransferase are identical to Figure 4 and shown for comparison only.

The process of synapse formation is thought to be mediated by the interaction of cell adhesion molecules located in the presynaptic and postsynaptic sites. Such is the case for presynaptic neurexin and postsynaptic neuroligin adhesion molecules, which have been implicated in synaptogenic activity and synapse maturation (Scheiffele *et al.* 2000; Graf *et al.* 2004; Südhof 2008). We found that *nrx-1*/neurexin null mutants exhibited defects in response to contact during male mating similar to the defects observed in *hst-3.1/*HS 3-*O*-sulfotransferase mutant worms (Figure 5A). *nlg-1*/neuroligin mutants, by contrast, did not show defects in response to contact behavior. To investigate whether *hst-3.1* still retained a function in the absence of *nrx-1* function, we constructed *hst-3.1*; *nrx-1* double mutants. We found that the *hst-3.1*; *nrx-1* double mutant further enhanced the defects observed in both single mutants, thus suggesting that these genes act in parallel genetic pathways (while not excluding the possibility that they act in the same pathway given the strong phenotype observed in the *hst-3.1/*HS 3-*O*-sulfotransferase single mutant) (Figure 5A). The *hst-3.1*; *nlg-1* double mutants suppressed the defect in response to contact during male mating observed in the *hst-3.1* single mutant, consistent with a parallel, *hst-3.1*-independent pathway (Figure 5A). We next examined *hst-3.1*; *nrx-1*; *nlg-1* triple-mutant worms in response behavior and observed that, in the absence of *nrx-1*/neurexin, *nlg-1*/neuroligin no longer suppresses the *hst-3.1*-dependent response defects, showing that *nrx-1*/neurexin is epistatic and suggesting that *nlg-1* acts to suppress the activity of *nrx-1*. To further investigate the *nlg-1*/neuroligin suppression of the HS-independent defects in response behavior, we constructed triple mutants for *nlg-1*/neuroligin and the two glypicans, *lon-2*/glypican and *gpn-1*/glypican, as *lon-2gpn-1* double mutants also showed defects in response behavior. The *nlg-1lon-2gpn-1* triple mutants did not show defects in response to contact, thus indicating that *nlg-1*/neuroligin also suppresses the defects of *lon-2gpn-1* double mutants, consistent with the suppression observed for *hst-3.1/*HS 3-*O*-sulfotransferase mutants (Figure 5A). Together, these results suggest that *nrx-1*/neurexin and *nlg-1*/neuroligin adhesion, possibly through opposing roles, are involved in promoting response behavior in a pathway parallel to that in which *hst-3.1/*HS 3-*O*-sulfotransferase and the glypicans act.

To determine whether the genetic interaction of these molecules is also regulating synapse formation, we examined the presynaptic levels of *mCherry*::*RAB-3* in B-type ray sensory neurons in the same series of *nrx-1*/neurexin and *nlg-1*/neuroligin double- and triple-mutant worms. The *hst-3.1*; *nrx-1* mutants showed an increased accumulation of *mCherry*::*RAB-3*; however, the increase in accumulation was not significantly different from *hst-3.1/*HS 3-*O*-sulfotransferase single-mutant worms (Figure 5B). Considering that *hst-3.1*; *nrx-1* double-mutant defects in behavior are significantly more severe than the defects in *hst-3.1/*HS 3-*O*-sulfotransferase single mutants, we conclude that either it is difficult to detect an even higher accumulation of *mCherry*::*RAB-3* as measured by the relative fluorescence densities, or that *nrx-1*-dependent behavioral defects result from a disruption in another synaptic connection.

Consistent with the behavioral results, the *hst-3.1*; *nlg-1* double mutant suppressed the *mCherry*::*RAB-3* accumulation in the presynaptic cell, decreasing it to control levels (Figure 5B). As we observed in response behavior, the *hst-3.1*; *nrx-1*; *nlg-1* triple mutants showed an increased accumulation of *mCherry*::*RAB-3* that was not suppressed by *nlg-1*/neuroligin, consistently indicating that, in the absence of *nrx-1*, *nlg-1* does not suppress the *hst-3.1/*HS 3-*O*-sulfotransferase-induced defects in B-type ray neuron presynaptic sites. Lastly, *nlg-1lon-2gpn-1* triple mutants showed *mCherry*::*RAB-3* levels comparable to those of control worms, again mirroring the genetic interactions observed for the male mating behavior.

## Discussion

How neuronal connectivity determines behavioral output in an organism remains one of the biggest questions in the neuroscience field. To address this question, one important aspect is to investigate the roles of molecules in the ECM that are involved in establishing and making these connections functional. In this work, we used male mating behavior in *C. elegans* as a readout of synaptic function, together with fluorescence labeling of synapses, to study the role of HS molecules in the formation of the male nervous system and its synaptic connectivity.

### HS molecules mediate male mating behavior in *C. elegans*

We found that *hst-3.1/*HS 3-*O*-sulfotransferase is acting in the same genetic pathway as *pkd-2/*polycystin-2, *lov-1/*polycystin-1, and *klp-6/*kinesin to regulate the response to contact behavior during male mating. However, the *hst-3.1/*HS 3-*O*-sulfotransferase focus of action is not the B-type ray neurons, but rather the downstream EF male-specific interneurons or the hypodermis. Based on the double-mutant defects, the HSPGs LON-2/glypican and GPN-1/glypican act in parallel to mediate response behavior. Since it has been shown that *gpn-1*/glypican is expressed in neurons (Hudson *et al.* 2006) and *lon-2*/glypican acts in the hypodermis to mediate different aspects of neuronal development (Pedersen *et al.* 2013), we propose that the response to contact behavior is mediated by 3-*O* sulfation to the HSs attached to GPN-1/glypican in neurons, and 3-*O* sulfation to the HSs attached to LON-2/glypican in the hypodermis. Even though glypicans possess a GPI anchor, it has been shown that LON-2/glypicans can be shed from epidermal cells, secreted, and diffused into the ECM, where they interact with components of Netrin signaling to mediate axon guidance (Blanchette *et al.* 2015).

Interestingly, only 3-*O*-sulfation by *hst-3.1/*HS 3-*O*-sulfotransferase, and not *hst-3.2/*HS 3-*O*-sulfotransferase, is regulating the mating process. The opposite specificity for the 3-*O* sulfotransferases was identified in the neurite branching induced due to overexpression of the cell adhesion molecule KAL-1/Anosmin 1 in the AIY interneuron (Bülow *et al.* 2002). In this context, the *kal-1*-induced branches in AIY were suppressed in the *hst-3.2* mutant, but not in the *hst-3.1/*HS 3-*O*-sulfotransferase mutant (Tecle *et al.* 2013), thus providing further evidence that both HS 3-*O*-sulfotransferase might display different substrate specificities or expression patterns (Moon *et al.* 2012).

It has been shown that the EF interneurons are important for male exploratory behavior, which is essential for males to localize to and contact their mating partners (Barrios *et al.* 2008). In terms of connectivity, their main synaptic input is from the B-type ray sensory neurons, while their main synaptic output is onto the AVB premotor interneuron (S. J. Cook, C. A. Brittin, T. A. Jarrell, Y. Wang, A. E. Bloniarz, personal communication). Previous cell ablation studies have shown that the EF interneurons mediate backward locomotion after mate contact (Sherlekar 2015), which is necessary for the response to contact behavior. Because AVB is the premotor interneuron that promotes forward movement in the locomotion circuit, while EF ablation promotes forward movement after mate contact, it is thought that the EF → AVB connections are inhibitory synapses. Our findings support this hypothesis, given that *hst-3.1/*HS 3-*O*-sulfotransferase is acting in the EFs to regulate response behavior, and the *hst-3.1/*HS 3-*O*-sulfotransferase mutants have defects in backward locomotion after contacting the hermaphrodite.

### HS 3-*O* sulfation regulates synapse formation of male mating neurons

Our findings, together with previously published studies, demonstrate that HSs are mediators of synapse development and function. In this work, we identify a HS motif with 3-*O*-sulfation likely attached to the HSPG LON-2/glypican and/or GPN-1/glypican, which is required for synaptogenesis. Our observations conceptually extend studies in cultured hippocampal neurons, where the LRRTM4’s synaptogenic activity requires the presence of HSs (de Wit *et al.* 2013), by establishing that specific HS modification patterns are important for this process. Moreover, they showed that glypican acts as a receptor for LRRTM4, and their interaction is important for the development of excitatory synapses.

Through the analysis of null mutations, we demonstrate that the elimination of *hst-3.1/*HS 3-*O*-sulfotransferase and the two glypican forms, *lon-2*/glypican and *gpn-1*/glypican, induces defects in the presynaptic specialization of B-type ray neurons, while it reduces the iBLINC synaptic labeling of RnBs → EF synapses, indicating their role in the process of synapse formation. The observation that the accumulation levels of the presynaptic marker RAB-3 in B-type ray neurons is higher in *hst-3.1/*HS 3-*O*-sulfotransferase mutants than in control worms suggests that vesicle fusion is not occurring properly at the synapse. The higher levels of RAB-3 can be explained by a change in the size and morphology of the synaptic puncta rather than an increase in their number. This is further supported by the fact that the presynaptic pattern of B-type ray neurons in the preanal ganglion of mutant worms is comparable to the one observed in control worms. In addition, using the iBLINC-labeling system, we showed that *hst-3.1/*HS 3-*O*-sulfotransferase mutants and *lon-2gpn-1* double mutants form fewer RnB → EFs synapses. In terms of synaptic function, from our results, we argue that the observed *hst-3.1/*HS 3-*O*-sulfotransferase and *lon-2gpn-1* defects in response behavior during mating are a reflection of the observed defects in synapse formation.

The HS 3-*O* sulfation regulation during the neuronal development of B-type ray sensory neurons seems to be specific for the process of synapse formation as no defects were observed in the axon morphology of these mating neurons. A similar role of HS molecules in the process of synapse formation has been previously reported in mammals, where ext1 conditional knockout in mice results in autism-like behavioral phenotypes due to abnormal functioning of glutamatergic synapses, while no detectable morphological defects were observed in the brain (Irie *et al.* 2012). Even though there is no involvement of HS 3-*O*-sulfation in the process of B-type ray neuron axon guidance, we found that other HS modifications such as 2-*O*-sulfation, 6-*O*-sulfation, and *C*-5 epimerization mediate anteroposterior and dorsoventral axon guidance pathways by acting in parallel genetic pathways. This is consistent with previous findings that demonstrate the variable function of distinct HS modification patterns in the neuronal developmental of different cell types (Saied-Santiago *et al.* 2017). The simultaneous knockdown of three proteoglycans, *sdn-1*/syndecan, *lon-2*/glypican, and *gpn-1*/glypican, severely affected the axon guidance of B-type ray neurons, demonstrating that they act redundantly in this process, as is the case for other processes such as KAL-1/Anosmin 1-induced neurite branching in the AIY interneuron (Díaz-Balzac *et al.* 2014). In the context of dorsoventral migration of B-type neurons, genetic elimination of HS molecules causes similar defects to *unc-6*/netrin ligand and its *unc-40*/DCC (Deleted in Colorectal Carcinoma) surface receptor, indicating that HSs may regulate axon guidance through the netrin signaling pathway. However, the mechanism by which this is accomplished remains elusive. Plausible possibilities include ligand sequestration, the modulation of ligand–receptor interaction, or a function of HSPGs as coreceptors (Blanchette *et al.* 2015; Díaz-Balzac *et al.* 2015; Poulain and Yost 2015). Future experiments should distinguish between these possibilities.

**Figure 6:**
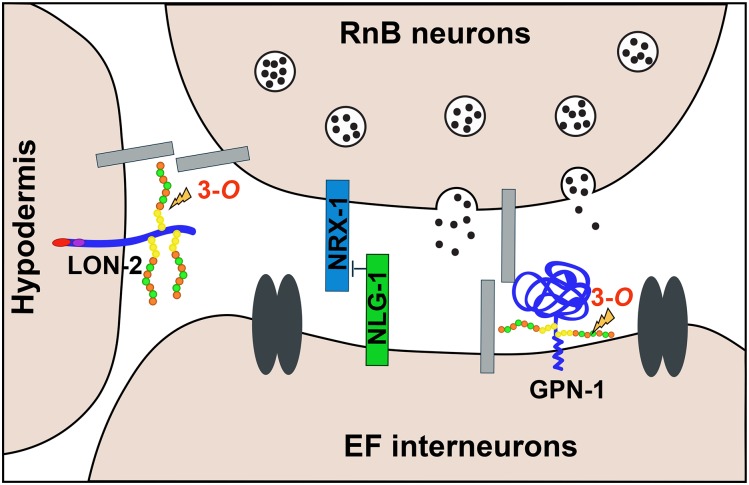
Proposed working model for the role of heparan sulfate (HS) 3-*O* sulfation in synapse formation. 3-*O* sulfation of HS chains located in LON-2 and GPN-1 mediates synapse formation between RnB neurons and EF interneurons, in parallel to NRX-1 and NLG-1, most likely by regulating the interaction between unidentified synaptic molecules. The absence of NRX-1 promotes synapse formation between RnB neurons and EF interneurons, while NLG-1 acts as a synaptic inhibitor. Disruption of this synaptic connection causes the accumulation of synaptic vesicles in the B-type ray neurons and behavioral defects in response to hermaphrodite contact during male mating. However, further biochemical experiments are needed to validate this model.

We have also shown that the neural cell adhesion proteins neurexin and neuroligin play a role in the formation of synapses by the RnB neurons. Neurexin promotes synapse formation while neuroligin opposes the function of neurexin. Interestingly, Hart and Hobert (2018) recently found a similar novel, antagonistic role for these proteins in synapse formation elsewhere in the *C. elegans* male mating circuits. This neurexin/neuroligin pathway is, to some extent, independent of the pathway in which *hst-3.1/*HS 3-*O*-sulfotransferase functions as each gene retains some function in null mutants of the other (Figure 6). In fact, in the absence of the inhibitory function of neuroligin, it appears that the synapse-promoting function of neurexin can fully restore synapse formation and function in the absence of *hst-3.1/*HS 3-*O*-sulfotransferase (Figure 5). These results indicating multiple independent pathways promoting synapse formation point out the complexity of the process and help us to understand how specificity and robustness are achieved.

## Supplementary Material

Supplemental material is available online at www.genetics.org/lookup/suppl/doi:10.1534/genetics.118.300837/-/DC1.
