# Supplementary material for: Synaptogenesis Is Modulated by Heparan Sulfate in Caenorhabditis elegans
Source: Genetics. 2018 Mar 20;209(1):195–208. doi: 10.1534/genetics.118.300837 (PMC5937176; doi:10.1534/genetics.118.300837)
Supplement: Supplementary file 4 [file 195FileS4.pdf]

Supplementary Material

to

Synaptogenesis is modulated by heparan sulfate in *C. elegans*

María I. Lázaro-Peña[1], Carlos A. Díaz-Balzac[1], Hannes E. Bülow[1,2], and Scott W. Emmons[1,2]*

Department of Genetics[1] and Dominick P. Purpura Department of Neuroscience[2]

Albert Einstein College of Medicine

Bronx, New York, 10461

* corresponding author:

Telephone 718 430 3130

Fax 718 430 8778

e-mail: scott.emmons@einstein.yu.edu

Running title: Heparan sulfate mediate synapse formation

6 Supplementary Figures

3 Supplementary Tables

3 Supplementary Files

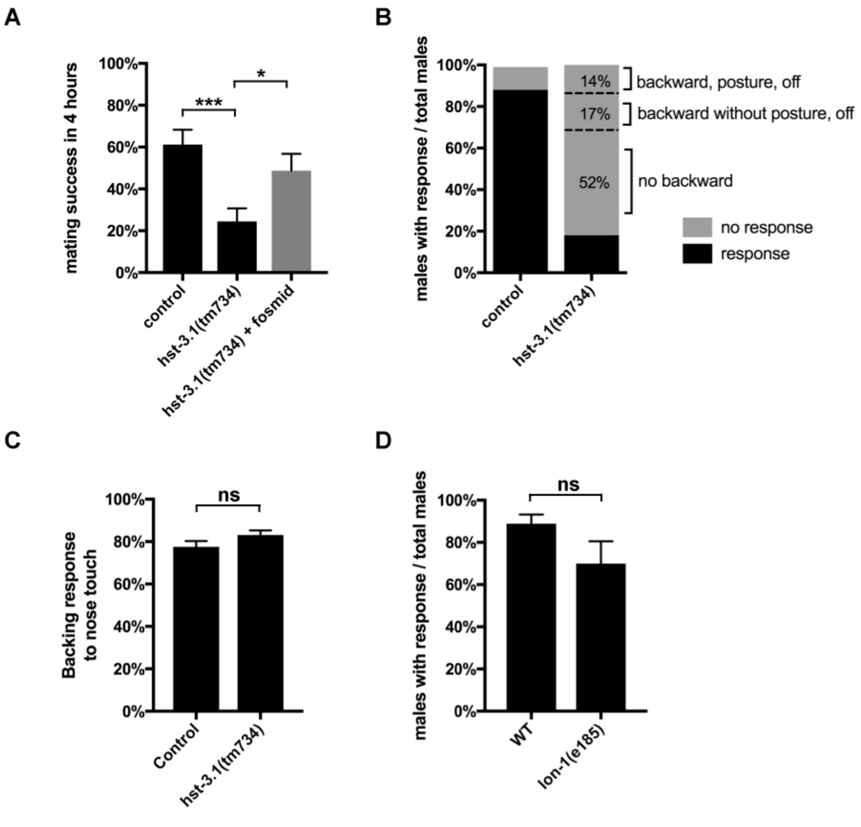

Figure S1 *hst-3.1*/HS 3-*O*-sulfotransferase male potency and quantification of defect

A) *hst-3.1*/HS 3-*O*-sulfotransferase loss of function affects mating success as less crossed progeny was observed. We let *hst-3.1(tm734)* mutant males mate with *pha-1(e2123)* mutant hermaphrodites for 4 hours and then looked at the progeny 2 days later. The *pha-1(e2123)* mutant worms are temperature sensitive and are not viable at 25°C, so only the *pha-1*/+ heterozygous cross progeny grow at 25°C. We counted and compared the crossed progeny of *hst-3.1(tm734)* versus control male worms and observed that only 24% of the *hst-3.1*/HS 3-*O*-sulfotransferase mutant males succeeded in mating compared to a 61% of the control males

B) Most of the non-responsive *hst-3.1(tm734)* males were not able to move backward after the tail contacted the hermaphrodite body. The other non-responsive *hst-3.1(tm734)*

mutant males showed a discontinued backward locomotion by swimming off in less than 2 seconds of contact.

C) *hst-3.1(tm734)* mutants did not show defects in backward locomotion after nose touch since the worms were able to move backward 83% of the times it was gently touched in the nose.

D) *lon-1(e185)* mutant worms did not show significant defects in response to hermaphrodite contact since 70% of mutant males responded to contact.

Error bars denote the SEM; statistical significance is shown as follows: $^{*}p < 0.05$; $^{**}p < 0.005$; $^{***}p < 0.0005$; $^{****}p < 0.00005$; and ns, not significant.

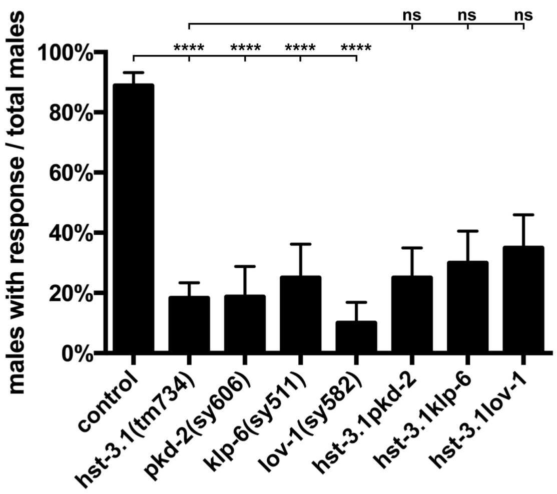

Figure S2 *hst-3.1/3-O sulfotransferase*, *pkd-2/*polycystin-2, *lov-1/*polycystin-1 and *klp-6/*kinesin act in the same genetic pathway.

Quantification of response to hermaphrodite contact during male mating behavior in the genotypes indicated. Error bars denote the SEM; statistical significance is shown as follows: $^{*}p < 0.05$; $^{**}p < 0.005$; $^{***}p < 0.0005$; $^{****}p < 0.00005$; and ns, not significant. The data for control and *hst-3.1* are identical to figure 1 and shown for comparison only.

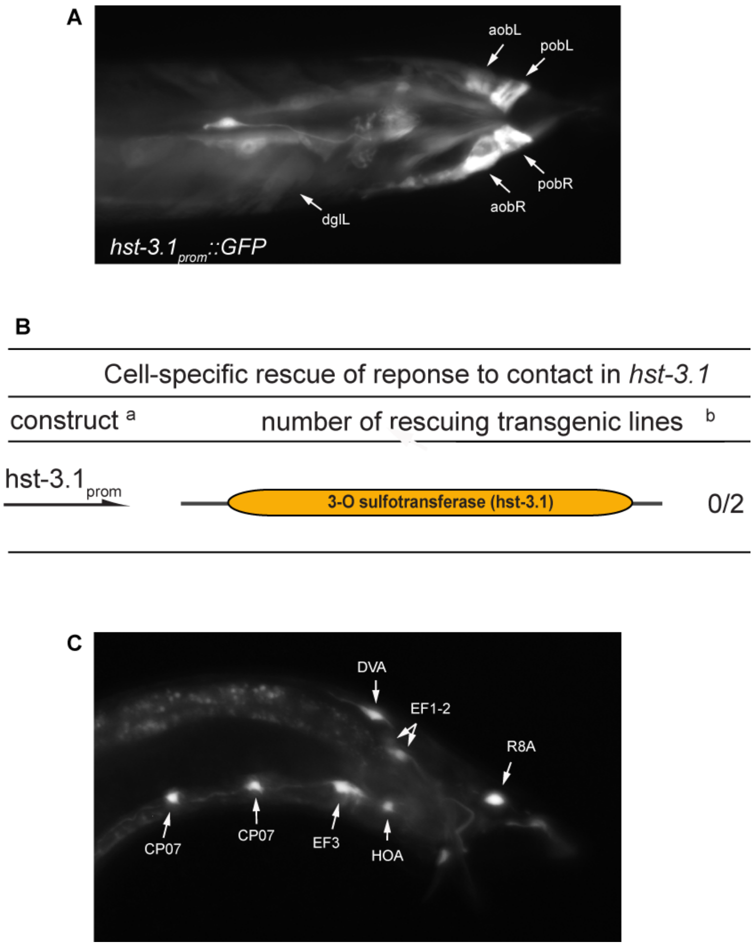

Figure S3 Transcriptional reporter of *hst-3.1*/HS 3-*O*-sulfotransferase and *nlg-1*/Neuroligin transcriptional GFP fusion of

A) Fluorescent ventral view of adult male animals showing the expression of the *hst-3.1p::GFP* transcriptional reporter containing 2,409 nt upstream the start codon (Tecle *et al.* 2013). In the male tail, expression is observed in male-specific muscles in the tail such as the oblique and diagonal muscles. Anterior is to the left.

B) Cell-specific rescue of response to hermaphrodite contact during male mating in *hst-3.1(tm734)* mutants. Schematically show is the construct/promoter used (*Phst-3.1)* and the number of rescuing lines of the total number of lines.

(C) Fluorescent lateral view of adult male animals showing the expression of the *nlg-1p::GFP* transcriptional fusion. In the male tail, expression is observed in the male-specific EF_{1-3} and CP_{7-8} interneurons, and R8A and HOA sensory neurons. Anterior is to the left.

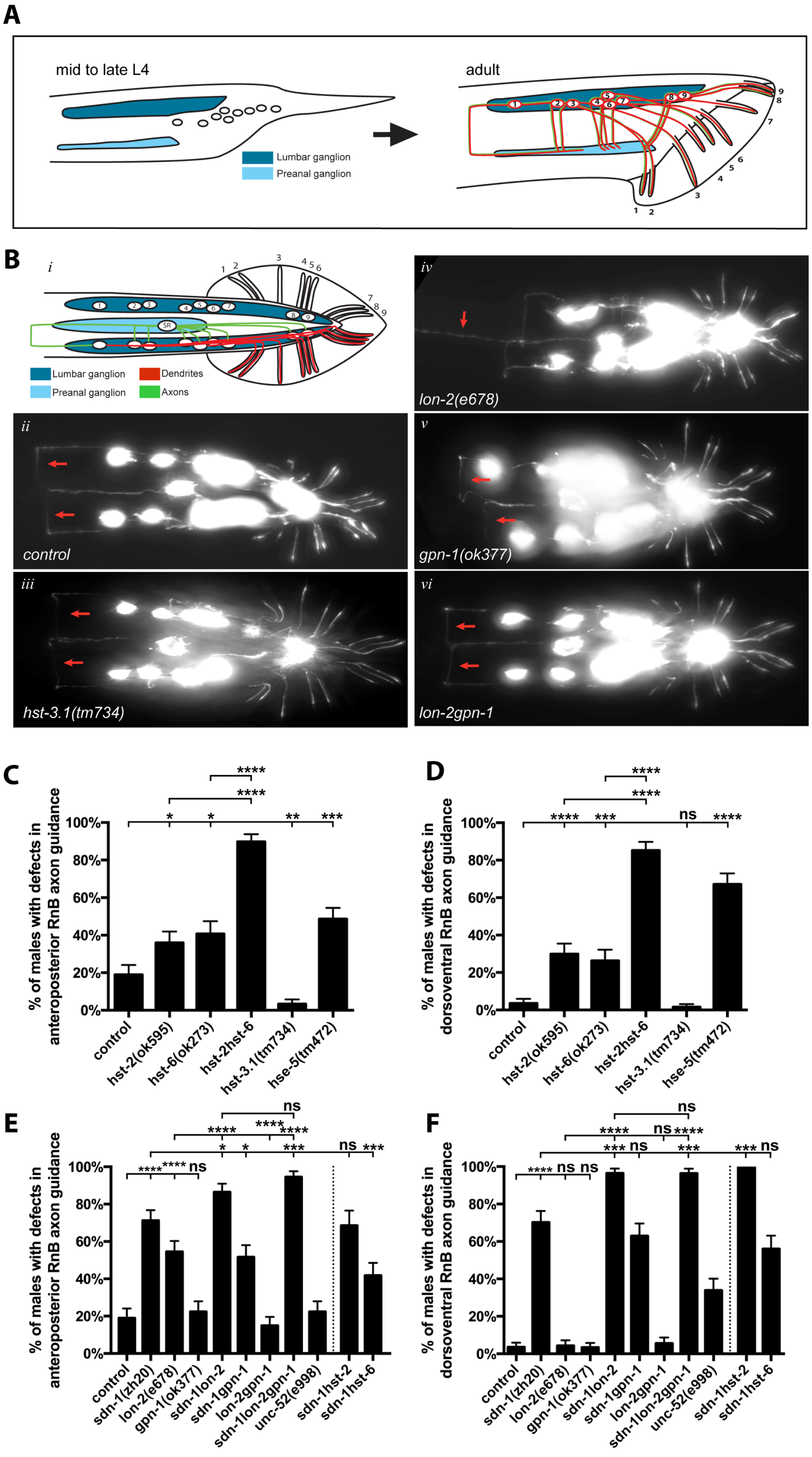

Figure S4 HSME and HSPG are required for axon guidance of B-type ray neurons

(A) Schematic of the development of A-type (Red) and B-type (Green) ray neurons in the posterior male nervous system. The dark blue represents the lumbar ganglion and the light blue represents the preanal ganglion location.

(B) Ventral views with schematics (*i-vi*) of adult male animals showing the B-type ray neurons. B-type ray neurons were visualized with *bxIs14* (*Is*[*Ppkd-2::GFP*]). Anterior is to the left.

(C–F) Quantification of B-type ray neurons anteroposterior and dorsoventral axon guidance in the genotypes indicated. Error bars denote the SEM; statistical significance is shown as follows: $^{*}p < 0.05$; $^{**}p < 0.005$; $^{***}p < 0.0005$; $^{****}p < 0.00005$; and ns, not significant. The data for control are identical (C-E and D-F) and shown for comparison only.

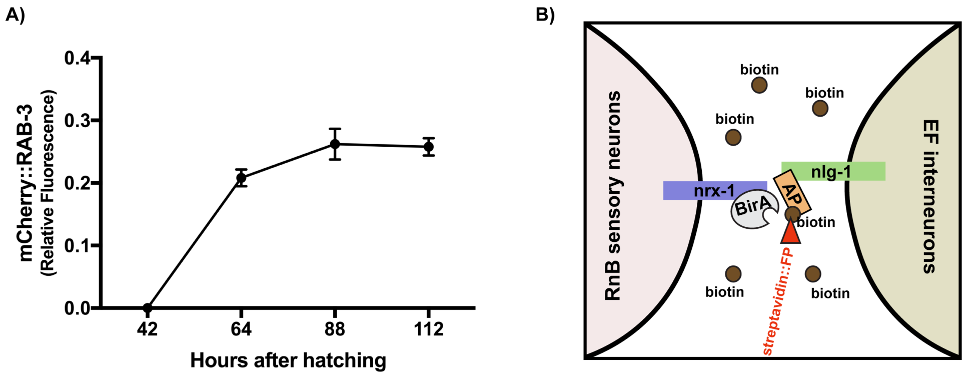

Figure S5 The development of B-type ray neuron synapses and a diagram of the iBLINC system used.

A) The presynaptic sites of B-type ray neurons were visualized and quantified using the *pkd-2p::mCherry::RAB-3* translational reporter during the course of time after egg hatching.

B) A diagram of the iBLINC trans-synaptic biotin transfer system showing the presynaptic BirA ligase fused N-terminally to NRX-1/neurexin in B-type ray sensory neurons and the postsynaptic acceptor peptide AP fused N-terminally to NLG-1/neuroligin in the EF interneurons (DESBOIS *et al.* 2015). Fluorescent streptavidin binds to biotin, labeling the synapses.

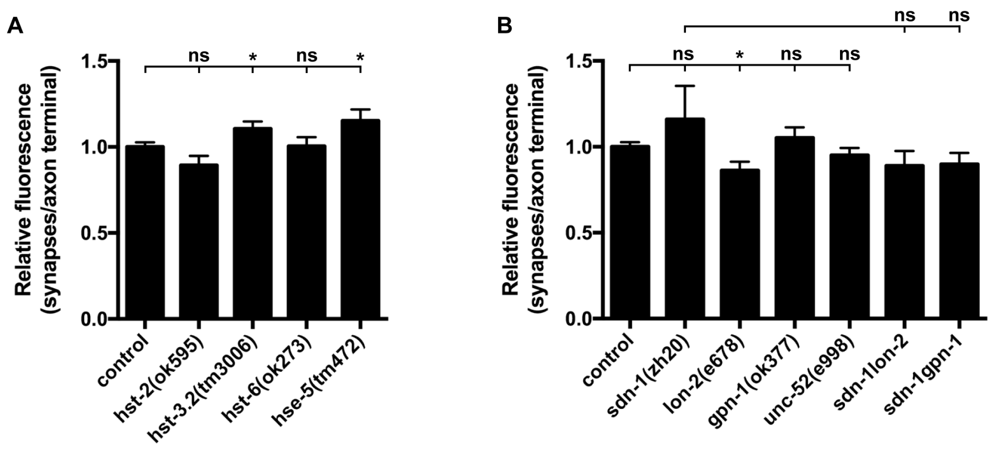

Figure S6 Presynaptic marker mCherry::RAB-3 quantification in the HSME and HSPG mutants.

(A-B) Quantification of mCherry::RAB-3 fluorescence in the preanal ganglion synaptic ring in the genotypes indicated. Error bars denote the SEM; statistical significance is shown as follows: $^{*}p < 0.05$; $^{**}p < 0.005$; $^{***}p < 0.0005$; $^{****}p < 0.00005$; and ns, not significant. The data for control are identical to figure 4 and shown for comparison only.

Table S1: List of all strains used in the study

Table S2: List of all transgenic strains used in the study

Table S3: List of all injection mixes used in the study

Table S1

**Strain name**	**Genotype**
EM1554	*bxIs30II; him-5(e1490)V*
EM1555	*bxIs30hst-3.1(tm734)II; him-5(e1490)V*
EM1556	*bxIs30II; him-5(e1490)V; hst-3.2(tm3006)X*
EM1557	*bxIs30II; him-5(e1490)V; hst-2(ok595)X*
EM1558	*bxIs30II; him-5(e1490)V; hst-6(ok273)X*
EM1559	*bxIs30II; hse-5(tm472)III; him-5(e1490)V*
EM1560	*bxIs30hst-3.1(tm734)II; him-5(e1490)V; hst-3.2(tm3006)X*
EM1561	*bxIs30II; him-5(e1490)V; sdn-1(zh20)X*
EM1562	*bxIs30II; him-5(e1490)V; lon-2(e678)X*
EM1563	*bxIs30II; him-5(e1490)V; gpn-1(ok377)X*
EM1564	*bxIs30II; him-5(e1490)V; lon-2(e678)gpn-1(ok377)X*
EM1565	*bxIs30IIhst-3.1 (tm734)II; him-5(e1490)V; lon-2(e678)gpn-1(ok377)X*
EM1566	*bxIs30IIhst-3.1 (tm734)II; him-5(e1490)V; sdn-1(zh20)lon-2(e678)X*
EM1567	*bxIs30IIhst-3.1 (tm734)II; him-5(e1490)V; sdn-1(zh20)gpn-1(ok377)X*
EM1568	*bxIs30II; him-5(e1490)V; nlg-1(ok259)X*
EM1569	*bxIs30IIhst-3.1(tm734)II; him-5(e1490)V; nlg-1(ok259)X*
EM1570	*bxIs30II; nrx-1(ok1649)him-5(e1490)V*
EM1571	*bxIs30IIhst-3.1(tm734)II; nrx-1(ok1649)him-5(e1490)V*
EM1572	*bxIs30II; pkd-2(sy606)IV; him-5(e1490)V*
EM1573	*bxIs30II; klp-6(sy511)III; him-5(e1490)V*
EM1574	*bxIs30IIlov-1(sy582)II; him-5(e1490)V*
EM1575	*bxIs30IIhst-3.1(tm734)II; pkd-2(sy606)IV; him-5(e1490)V*
EM1576	*bxIs30IIhst-3.1(tm734)II; klp-6(sy511)III; him-5(e1490)V*

EM1577	*bxIs30IIhst-3.1(tm734)lov-1(sy582)II; him-5(e1490)V*
EM733	*bxIs14him-5(e1490)V*
EM1578	*hst-3.1(tm734)II; bxIs14him-5(e1490)V*
EM1579	*bxIs14him-5(e1490)V; hst-2(ok595)X*
EM1580	*bxIs14him-5(e1490)V; hst-6(ok273)X*
EM1581	*hse-5(tm472)III; bxIs14him-5(e1490)V*
EM1582	*bxIs14him-5(e1490)V; hst-2(ok595)hst-6(ok273)X*
EM1583	*bxIs14him-5(e1490)V; sdn-1(zh20)X*
EM1584	*bxIs14him-5(e1490)V; lon-2(e678)X*
EM1585	*bxIs14him-5(e1490)V; gpn-1(ok377)X*
EM1586	*bxIs14him-5(e1490)V; lon-2(e678)sdn-1(zh20)X*
EM1587	*bxIs14him-5(e1490)V; lon-2(e678)gpn-1(ok377)X*
EM1588	*bxIs14him-5(e1490)V; sdn-1(zh20)gpn-1(ok377)X*
EM1618	*hst-3.1(tm734)II; bxIs14him-5(e1490)V; bxEx376*
EM1619	*bxIs14him-5(e1490)V; lon-2(e678)gpn-1(ok377)X; bxEx376*
EM1620	*bxIs30II; nrx-1(ok1649)him-5(e1490)V; lon-2(e678)X*
EM1621	*bxIs30II; nrx-1(ok1649)him-5(e1490)V; lon-2(e678)gpn-1(ok377)X*
EM1622	*bxIs30II; him-5(e1490)V; lon-2(e678)nlg-1(ok259)X*
EM1623	*bxIs30II; nrx-1(ok1649)him-5(e1490)V; nlg-1(ok259)X*
EM1624	*unc-52(e998)II; bxIs14him-5(e1490)V*
EM1625	*bxIs14him-5(e1490)V; sdn-1(zh20) hst-2(ok595)X*
EM1626	*bxIs14him-5(e1490)V; sdn-1(zh20)hst-6(ok273)X*
EM1627	*hse-5(tm472)III; bxIs14him-5(e1490)V; sdn-1(zh20)X*
EM1628	*bxIs14him-5(e1490)V; unc-6(ev400)X*
EM1629	*unc-40(e271)I; bxIs14him-5(e1490)V*
EM1670	*bxIs30II; him-5(e1490)V; lon-1(185)III*

Table S2

Strain name	Injection name	Genotype
EM1589	ml44	*bxIs30II; hst-3.1(tm734)III; him-5(e1490)V; bxEx348*
EM1590	ml21	*bxIs30II; hst-3.1(tm734)III; him-5(e1490)V; bxEx349*
EM1591	ml21	*bxIs30II; hst-3.1(tm734)III; him-5(e1490)V; bxEx350*
EM1592	ml22	*bxIs30II; hst-3.1(tm734)III; him-5(e1490)V; bxEx351*
EM1593	ml22	*bxIs30II; hst-3.1(tm734)III; him-5(e1490)V; bxEx352*
EM1594	ml22	*bxIs30II; hst-3.1(tm734)III; him-5(e1490)V; bxEx353*
EM1595	ml23	*bxIs30II; hst-3.1(tm734)III; him-5(e1490)V; bxEx354*
EM1596	ml23	*bxIs30II; hst-3.1(tm734)III; him-5(e1490)V; bxEx355*
EM1597	ml23	*bxIs30II; hst-3.1(tm734)III; him-5(e1490)V; bxEx356*
EM1598	ml23	*bxIs30II; hst-3.1(tm734)III; him-5(e1490)V; bxEx357*
EM1599	ml28	*bxIs30II; hst-3.1(tm734)III; him-5(e1490)V; bxEx358*
EM1600	ml28	*bxIs30II; hst-3.1(tm734)III; him-5(e1490)V; bxEx359*
EM1671	ml28	*bxIs30II; hst-3.1(tm734)III; him-5(e1490)V; bxEx385*
EM1601	ml29	*bxIs30II; hst-3.1(tm734)III; him-5(e1490)V; bxEx360*
EM1602	ml29	*bxIs30II; hst-3.1(tm734)III; him-5(e1490)V; bxEx361*
EM1672	ml29	*bxIs30II; hst-3.1(tm734)III; him-5(e1490)V; bxEx386*
EM1603	ml39	*bxIs30II; hst-3.1(tm734)III; him-5(e1490)V; bxEx362*
EM1604	ml39	*bxIs30II; hst-3.1(tm734)III; him-5(e1490)V; bxEx363*
EM1673	ml39	*bxIs30II; hst-3.1(tm734)III; him-5(e1490)V; bxEx387*
EM1605	ml37	*bxIs30II; hst-3.1(tm734)III; him-5(e1490)V; bxEx364*
EM1606	ml37	*bxIs30II; hst-3.1(tm734)III; him-5(e1490)V; bxEx365*
EM1674	ml37	*bxIs30II; hst-3.1(tm734)III; him-5(e1490)V; bxEx388*

EM1607	ml35	*bxIs30II; hst-3.1(tm734)III; him-5(e1490)V; bxEx366*
EM1608	ml35	*bxIs30II; hst-3.1(tm734)III; him-5(e1490)V; bxEx367*
EM1675	ml35	*bxIs30II; hst-3.1(tm734)III; him-5(e1490)V; bxEx389*
EM1609	ml36	*bxIs30II; hst-3.1(tm734)III; him-5(e1490)V; bxEx368*
EM1610	ml36	*bxIs30II; hst-3.1(tm734)III; him-5(e1490)V; bxEx369*
EM1611	ml36	*bxIs30II; hst-3.1(tm734)III; him-5(e1490)V; bxEx370*
EM1612	ml38	*bxIs30II; hst-3.1(tm734)III; him-5(e1490)V; bxEx371*
EM1613	ml38	*bxIs30II; hst-3.1(tm734)III; him-5(e1490)V; bxEx372*
EM1614	ml38	*bxIs30II; hst-3.1(tm734)III; him-5(e1490)V; bxEx373*
EM1615	ml33	*bxIs30II; hst-3.1(tm734)III; him-5(e 1490)V; bxEx374*
EM1616	ml33	*bxIs30II; hst-3.1(tm734)III; him-5(e1490)V; bxEx375*
EM1617	ml42	*bxIs14him-5(e1490)V; bxEx376*
EM1676	ml45	*bxIs30II; hst-3.1(tm734)III; him-5(e1490)V; bxEx390*
EM1677	ml45	*bxIs30II; hst-3.1(tm734)III; him-5(e1490)V; bxEx391*
EM1678	ml45	*bxIs30II; hst-3.1(tm734)III; him-5(e1490)V; bxEx392*

Table S3

**Injection name**	**Main plasmid**	**Conc.**	**Marker**	**Conc.**	**Injected into**
ml44	WRM066cE09	50ng/μl	unc-122::mCherry	50 ng/μl	EM1555
ml21	prgef-1::hst-3.1	5ng/μl	ceh-22::gfp	40ng/μl	EM1555
ml22	pmyo-3::hst-3.1	5ng/μl	ceh-22::gfp	40ng/μl	EM1555
ml23	pdpy-7::hst-3.1	5ng/μl	ceh-22::gfp	40ng/μl	EM1555
ml28	ppkd-2::hst-3.1	5ng/μl	ceh-22::gfp	50ng/μl	EM1555
ml29	ptph-1::hst-3.1	5ng/μl	ceh-22::gfp	50ng/μl	EM1555

ml30	prgef-1::hst-3.2	5ng/μl	ceh-22::gfp	50ng/μl	EM1555
ml33	phst-3.1::hst-3.1	5ng/μl	ceh-22::gfp	50ng/μl	EM1555
ml35	pnlp-14::hst-3.1	5ng/μl	ceh-22::gfp	50ng/μl	EM1555
ml36	pnmr-1::hst-3.1	5ng/μl	ceh-22::gfp	50ng/μl	EM1555
ml37	peat-4::hst-3.1	5ng/μl	ceh-22::gfp	50ng/μl	EM1555
ml38	pnlg-1::hst-3.1	5ng/μl	ceh-22::gfp	50ng/μl	EM1555
ml39	pcat-2::hst-3.1	5ng/μl	ceh-22::gfp	50ng/μl	EM1555
ml42	ppkd-2::BirA::nrx-1 + pnlg-1::AP::nlg-1 + punc-122::strep::RFP	25ng/μl: 25ng/μl: 25ng/μl	ttx-3::gfp	25ng/μl	EM733
ml45	punc-47::hst-3.1	5ng/μl	ceh-22::gfp	50ng/μl	EM1555

File S1: Raw data of response to contact assays

File S2: Raw data of mCherry::RAB-3 and iBlinc fluorescence assays

File S3: Raw data of RnB sensory neurons axon guidance
